# Sequelae of a Rare Case of Penetrating Parotid Gland Injury: Ultrasound and Magnetic Resonance Imaging Features

**DOI:** 10.7759/cureus.19630

**Published:** 2021-11-16

**Authors:** Parag S Mahajan, Hatem Abdulmajeed, Abdulmalek Aljafari, Jouhar J Kolleri

**Affiliations:** 1 Clinical Imaging Department, Hamad Medical Corporation, Doha, QAT; 2 Internal Medicine, Al Neelain University, Khartoum, SDN

**Keywords:** foreign body, truama, magnetic resonance imaging, ultrasound, penetrating parotid gland injury, parotid gland injury

## Abstract

Parotid gland trauma is a rare condition due to the anatomical location of the parotid gland. Imaging of the parotid gland trauma is restricted to ultrasound (US), sialogram, and computed tomography scans in the published literature. We present a case of penetrating parotid gland injury caused by a fishhook. The foreign object was removed under local anesthesia and the patient was managed conservatively with antibiotics and tetanus toxoid vaccine. A swelling appeared in the left parotid region after two weeks that was diagnosed using US and magnetic resonance imaging as granulation tissue formation in the injured parotid gland.

## Introduction

Trauma to the parotid gland is a rare condition usually caused by a penetrating injury or a fracture of the facial skeleton. Patients often present with facial swelling, skin laceration, or bruising at the site of injury. Due to its low incidence, the diagnosis of parotid gland injury can be delayed leading to morbidities that include facial scarring, sialocele formation, salivary-cutaneous fistula formation, other short and long-term complications [1-3]. Investigators suggest that appropriate treatment and repair should be provided as early as possible, to avoid such complications [4].

We present an unusual case of penetrating injury to the parotid gland caused by a fishhook that pierced the auricle of the left ear and penetrated the subcutaneous soft tissue of the upper neck behind the left auricle. We discuss the radiological findings, management and follow-up of this patient.

## Case presentation

**Figure 1:**
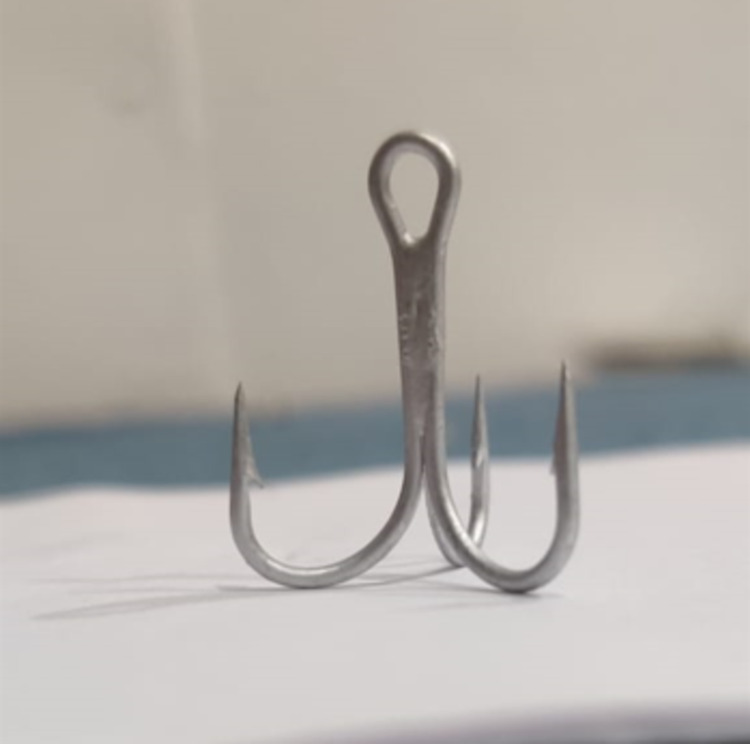
Triple needle fishhook

A 35-year-old male presented with a foreign body in his left ear caused by a trauma to the left parietal area by a fishhook. The patient was in a boat on a fishing trip when the fishhook accidentally pierced his upper neck behind the left auricle and pierced the auricle of the left ear. On examination, the patient was conscious and oriented and no bleeding, swelling, hematoma or bruises were noticed. Vital measurements and systemic review revealed normal findings. The patient received an intramuscular injection of 0.5 mL tetanus toxoid adsorbed vaccine and was referred for surgical assessment and foreign body removal. Under local anesthesia, the triple needle fishhook was removed and cut by a bone nipper from left pinna and post-auricular area (Figure 1). The lacerated wound was stitched by 05 Ethilon suture, left mastoid dressing was applied and the patient was discharged after prescribing per-oral cefuroxime and diclofenac for five days.

Two weeks later, the patient returned to the hospital for follow-up. On examination, left pinna and post-auricular area were normal. However, a small, non-tender, firm 2 x 2 mm subcutaneous swelling was noticed below the ear lobule. Amoxicillin/clavulanate and diclofenac sodium were prescribed, and the patient was discharged.

**Figure 2:**
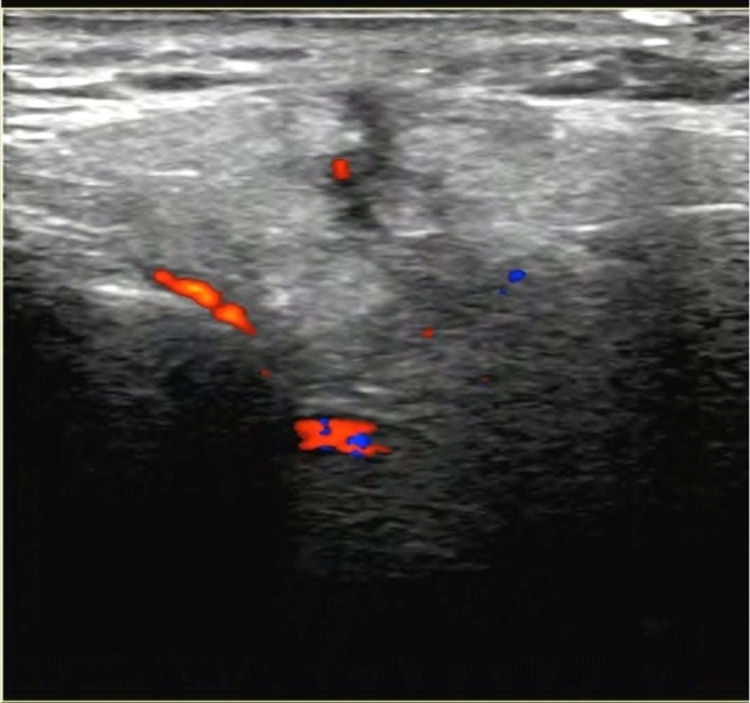
Ultrasound scan of the parotid gland shows a linear hypoechoic focus in the superficial parotid gland extending to the subcutaneous tissue

**Figure 3:**
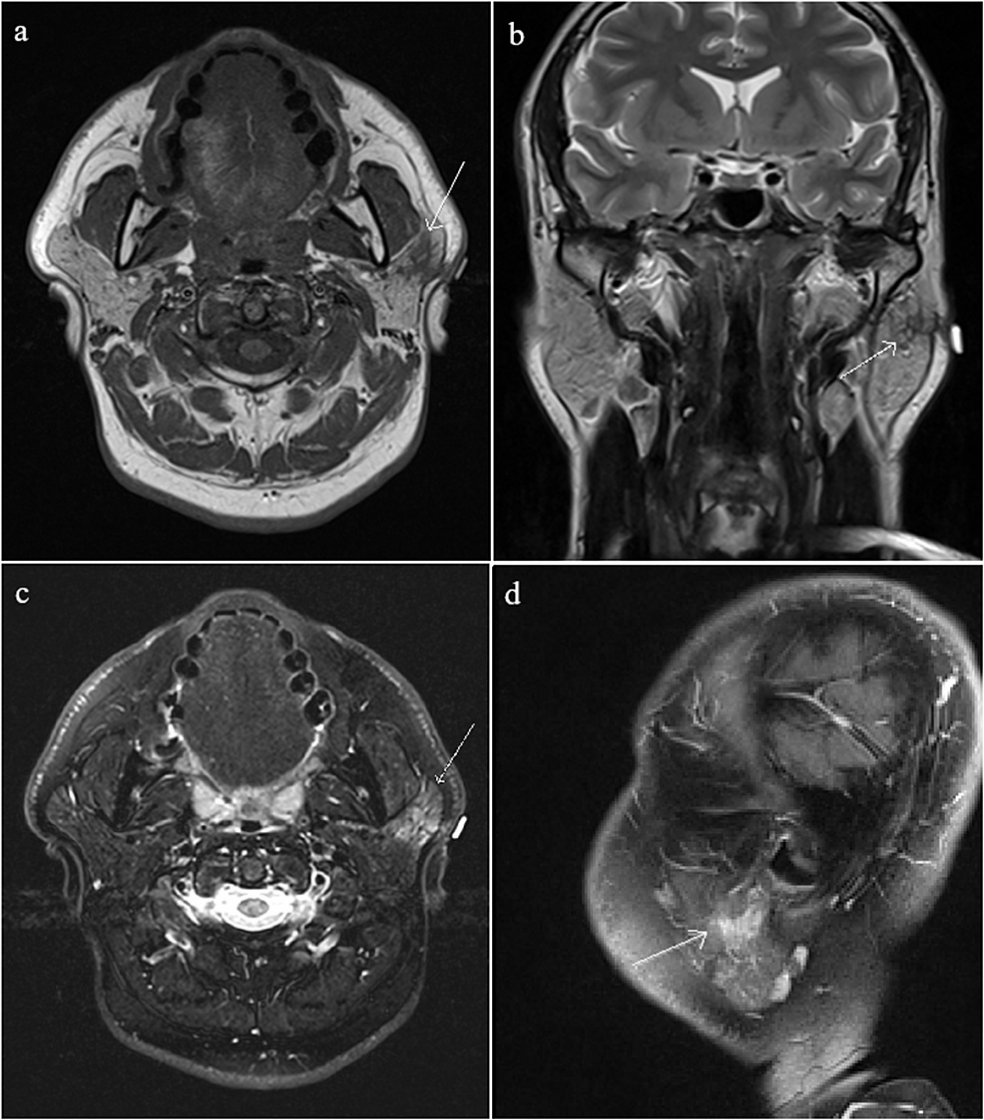
(a) T1-weighted axial and (b) T2-weighted coronal images show an ill-defined linear branching T1 and T2-hypointense area in the area of concern involving the superficial part of the left parotid gland, extending to the subcutaneous soft tissue with adjoining T2-hyperintense areas. (c) Axial short tau inversion recovery (STIR) and (d) sagittal post-contrast T1-weighted images show enhancement along the linear branching area

In the follow-up visit two months after the injury, the patient was assessed for a localized small, non-tender, 2 x 2 mm parotid swelling at the angle of mandible on the left side. The swelling appeared after the removal of the foreign body two months ago and did not get resolved. Ultrasound (US) of the neck showed a linear hypoechoic focus in the superficial parotid gland extending to the subcutaneous tissue (Figure 2). The presence of scar or granulation tissue was suspected and no focal mass lesions were detected. In addition, a few oval-shaped, non-specific cervical lymph nodes were noted with preserved fatty hilum and a short axis diameter of 0.8 cm. Magnetic resonance imaging (MRI) confirmed the presence of a post-traumatic scar and granulation tissue involving the superficial part of the left parotid gland and extending to the subcutaneous soft tissue (Figure 3). The presence of bilateral non-specific lymph nodes was noted in both cervical regions.

## Discussion

Parotid gland trauma is rare and most commonly caused by a penetrating injury by a knife or a sharp object. It may be associated with damage to the adjacent structures such as the facial nerve, the ear and bony structures of the face [5,6]. With the deep part of the parotid gland being relatively protected, most injuries often involve the superficial part of the gland [1,2]. Injuries to the branch duct and main duct sparing the parotid gland parenchyma are also possible [2]. To the best of our knowledge, this is the first case of a parotid gland injury by a fishhook to be reported. Also, to our knowledge, the US and MRI features of sequelae of the penetrating parotid gland injury have not been reported in the literature till date.

Parotid gland injury can be complicated by a variety of different outcomes depending on the severity and mode of injury, in addition to the quality of management. Lewis and Knottenbelt reported complications including salivary fistulas and sialoceles. One patient initially had a fistula, which was closed but eventually formed a sialocele. Another patient who developed a fistula ended up with wound sepsis that was treated with amoxicillin. No clinical complications were reported in many cases [2]. Although our patient experienced the formation of granulation tissue in the parotid gland and damage to the left ear, no ductal injury, fistula or sialocele complicated the injury.

The treatment and management of parotid gland injuries and complications vary from case to case, with conservative management being preferred as long as surgical intervention is not a necessity [5,7]. In our case, with the foreign body penetrating and damaging the left ear, a surgical intervention was necessary as an emergency. Although our patient received immediate and appropriate management, that did not prevent the formation of granulation tissue. In a prospective study on 51 cases, Parekh et al. concluded that conservative treatment had excellent prognosis in glandular and parotid duct injuries [1]. They found that only after adequate conservative treatment of a persistent fistula or sialocele, surgical intervention could be considered. Lewis and Knottenbelt recommended avoidance of surgical interventions, as its role was not justified and concluded that non-operative management should be enough for all parotid ductal injuries [2].

## Conclusions

Considering the low incidence of parotid gland injuries, it is important not to miss the diagnosis to avoid hazardous outcomes and to provide the best possible treatment. With complications including fistulas, sialoceles and scars, physicians should be alert and conscious when dealing with a suspected involvement of the parotid. With knives and sharp objects being the most common cause of injury, one must not miss a diagnosis when less common objects, such as a fishhook, are involved.
